# Understanding health behavior change by motivation and reward mechanisms: a review of the literature

**DOI:** 10.3389/fnbeh.2023.1151918

**Published:** 2023-06-19

**Authors:** Maren M. Michaelsen, Tobias Esch

**Affiliations:** Institute for Integrative Health Care and Health Promotion, Faculty of Health, Witten/Herdecke University, Witten, Germany

**Keywords:** motivation and reward, reinforcement processes, positive affect, reflective resources, nudging, boosting, facilitating, health behavior change

## Abstract

The global rise of lifestyle-related chronic diseases has engendered growing interest among various stakeholders including policymakers, scientists, healthcare professionals, and patients, regarding the effective management of health behavior change and the development of interventions that facilitate lifestyle modification. Consequently, a plethora of health behavior change theories has been developed with the intention of elucidating the mechanisms underlying health behavior change and identifying key domains that enhance the likelihood of successful outcomes. Until now, only few studies have taken into account neurobiological correlates underlying health behavior change processes. Recent progress in the neuroscience of motivation and reward systems has provided further insights into the relevance of such domains. The aim of this contribution is to review the latest explanations of health behavior change initiation and maintenance based on novel insights into motivation and reward mechanisms. Based on a systematic literature search in PubMed, PsycInfo, and Google Scholar, four articles were reviewed. As a result, a description of motivation and reward systems (approach/wanting = pleasure; aversion/avoiding = relief; assertion/non-wanting = quiescence) and their role in health behavior change processes is presented. Three central findings are discussed: (1) motivation and reward processes allow to distinguish between goal-oriented and stimulus-driven behavior, (2) approach motivation is the key driver of the individual process of behavior change until a new behavior is maintained and assertion motivation takes over, (3) behavior change techniques can be clustered based on motivation and reward processes according to their functional mechanisms into facilitating (= providing external resources), boosting (= strengthening internal reflective resources) and nudging (= activating internal affective resources). The strengths and limitations of these advances for intervention planning are highlighted and an agenda for testing the models as well as future research is proposed.

## 1. Introduction

The prevalence of lifestyle-related chronic diseases has increased dramatically in the last decades. Chronic diseases were responsible for 71% of all deaths occurring worldwide in 2019 (World Health Organisation [WHO], 2022a), of which about one third are premature deaths, i.e., happening to people aged between 30 and 69 years (World Health Organisation [WHO], 2022a). Diseases of the circulatory system like stroke and ischaemic heart disease accounted for 30% of all deaths in 2019 in OECD countries, followed by cancer (24%), diseases of the respiratory system (10%) and diabetes (3%) (Organisation for Economic Co-operation and Development [OECD], 2021). Individuals living with these conditions also face a major stress burden due to disability, in some cases already at young ages. Indeed, averaged across 26 OECD countries, more than one third of individuals aged 16 and over have been found to be living with longstanding illness or health problems (Organisation for Economic Co-operation and Development [OECD], 2021). In addition, comorbidities (multimorbidity) as well as individual physical and emotional suffering frequently occur (Stewart et al., 1989; Moussavi et al., 2007; de Ridder et al., 2008), reducing overall quality of life (Maresova et al., 2019).

These numbers and trends can in part be traced back to rising rates of obesity, sedentary behavior and poor nutrition, as well as other metabolic risk factors for chronic diseases including tobacco use and harmful alcohol intake. In addition, as diseases and comorbidities accumulate in older age, countries’ aging populations further influence these numbers (Zhou et al., 2016). Indeed, most countries in the world have experienced, and will experience great demographic transitions. It has been estimated that between 2015 and 2050, the number of individuals aged 60 years and older will nearly double from 12 to 22%, with two billion people aged above 60 years by 2050 (World Health Organisation [WHO], 2022b). At the same time, life expectancy has risen from 67.5 years in 2000 to 72.9 years in 2020 at the world’s average (The World Bank, 2022). Based on these projections, it can be assumed that the total number of individuals with longstanding illnesses or health problems will continue to rise.

The treatment of chronic diseases is often lengthy and intense, and is frequently accompanied by a reduced ability to work (Seuring et al., 2015). While this can reduce the quality of life in patients further (Jing et al., 2018), it can also affect an individual’s household financial resources (Seuring et al., 2015). In low income settings, tremendous costs for treatment can quickly drain savings (World Health Organisation [WHO], 2022a). This, in return, may perpetuate people’s conditions, as it has been found that poverty is closely linked with the prevalence of chronic diseases: vulnerable and socially disadvantaged people tend to get ill quicker and have lower life expectancy than people of higher social positions (World Health Organisation [WHO], 2022a). The main reasons for this phenomenon are that economically vulnerable individuals are at greater risk of being exposed to harmful products, such as tobacco, tend to have unhealthy diets, and, in some countries, cities or neighborhoods, have limited access to health services. In fact, the average life expectancy at birth of people with low income is 4.4 (women) to 8.6 (men) years lower than of people in the highest of five income groups (Lampert et al., 2019).

These costs on individuals are accompanied by costs for the healthcare system and society as a whole. Health expenditure related to diabetes, for example, is at least 966 billion USD per year worldwide, which represents a 316% increase over the last 15 years (International Diabetes Federation [IDF], 2021). In Germany, the cost burden for diabetes type 2 treatment has been calculated to be on average 1.8 times higher than for other diseases (Ulrich et al., 2016). Multimorbidity typically incurs greater health care costs (Rizzo et al., 2015), measured by the use of medication as well as emergency department presentations and hospital admissions (Chan et al., 2002). For example, Schneider et al. (2009) found that older adults in the United States with three or more chronic conditions utilized on average 25 times more hospital bed-days and had on average 14.6 times more hospital admissions than older adults without any chronic condition. Furthermore, with one additional chronic condition in older adults, the health care utilization costs increase near exponentially (Lehnert et al., 2011). In addition to these financial impacts, chronic conditions tend to dwell on non-tangible resources, e.g., through time and energy spent on disease management by the patient and family members (Ellrodt et al., 1997; Korff et al., 1998; Wagner, 2000). These circumstances call for shifting the focus to health care measures that help to prevent and improve chronic conditions according to patient needs in a cost-effective way.

There is compelling evidence to suggest that lifestyle changes can significantly improve the conditions of chronic diseases. Studies have demonstrated the positive impact of increased exercise, healthier nutrition, reduced alcohol intake, smoking cessation, and relaxation techniques on a range of chronic conditions (Ornish et al., 1990; Knowler et al., 2002; Savoye et al., 2007; Alert et al., 2013; Cramer et al., 2014; Morris et al., 2019). These health behaviors can decrease the major metabolic risk factors for chronic diseases and premature deaths, including blood pressure, blood glucose, blood lipids, and obesity (World Health Organisation [WHO], 2022a). Remarkably, the risk of developing type 2 diabetes is predominantly attributable to lifestyle-related factors rather than genetic risks (Langenberg et al., 2014). Moreover, lifestyle changes could prevent up to 70% of strokes and cases of colon cancer, 80% of coronary heart diseases, and 90% of diabetes cases (Willett, 2002). Such findings highlight the tremendous potential of lifestyle modification interventions for public health outcomes.

It is widely recognized that individuals encounter challenges when endeavoring to attain their lifestyle goals. This is not unexpected, given that lifestyle change necessitates a series of individual choices that often require postponement of immediate pleasure in favor of prospective long-term health gains (a.k.a. delayed gratification, present bias, hyperbolic discounting, etc., see Stroebe et al., 2008, 2013; Hall and Fong, 2015). Despite these obvious difficulties, practitioners, politicians and stakeholders aim to engage patients in health behavior change (Esch, 2018). How consistently individuals pursue health behavior changes depends largely on how well they can overcome their innate present bias and on their endowment with other resources, such as their knowledge about health behavior change consequences, their beliefs in their ability to succeed, their self-regulation skills, self-efficacy, internal locus of control, engagement and empowerment (Cane et al., 2012; Cheng et al., 2016; Sheeran et al., 2016; Ludwig et al., 2020; Cardoso Barbosa et al., 2021). Hence, a thorough understanding of health behavior change and interventions to support health behavior change taking into account individuals’ resources are necessary.

Numerous health behavior change theories have been devised, with a primary emphasis on reflective resources and willpower (Kwasnicka et al., 2016). However, there is a scarcity of research on domains that are supported by, or rooted in, neuroscientific evidence. Notably, recent advances in the neuroscience of motivation and reward systems have revealed new insights into the importance of such domains (Michaelsen and Esch, 2021, 2022).

The aim of this contribution is to provide an overview of the latest explanations of health behavior change initiation and maintenance based on novel insights to motivation and reward mechanisms. Based on a literature search in PubMed (22 hits), PsycInfo (39 hits), and Google Scholar using the term “motivation AND reward AND (‘behavior change’ OR ‘behavior modification’)” in titles and abstracts in January 2023, we identified four articles which discuss neurobiological mechanisms of reward and motivation in relation to health behavior change (Letzen et al., 2019; Ludwig et al., 2020; Michaelsen and Esch, 2021, 2022). These are integrated into the social psychological literature on behavior change, previously reviewed in Michaelsen and Esch (2021, 2022). The review is structured as follows: the next chapter presents a summary of behavior change theories as discussed in social and health psychology in order to provide thorough ground for the discussion of the role of motivation and reward processes in health behavior change. This is followed by a description of motivation and reward processes as recently discussed in neurobiological science. After this, three models are presented which take into account motivation and reward mechanisms in health behavior change and thereby combine the two strands of literature and present interesting avenues for future health behavior change intervention planning and implementation. A discussion of the review and future research is presented at the end of the article.

## 2. Behavior change theories in social and health psychology

A large number of theories aiming to explain health behavior change have been published in recent decades, most of them grounded in social and health psychology. These theories differ in the views of human nature they hold (Bandura, 1989) as well as in what they consider to be the fundamental drivers of behavior and the resources necessary for behavior change.

Established theories are concerned with the determinants of and motives for initiation of behavior change, and some also take into account the domains that enhance the likelihood of maintaining a new behavior after initiation (Kwasnicka et al., 2016). Among the leading theories are Bandura’s Social Cognitive Theory (Bandura, 1989), Gollwitzer’s theory on Implementation Intentions (Gollwitzer, 1999), and the Social Determination Theory by Ryan and Deci (2000) and Deci and Ryan (2008). In Bandura’s Social Cognitive Theory, individuals are assumed to learn new behaviors not only through trial-and-error but also through copying the behavior of others. Based on the existence of role models, the performance of the new behavior is enhanced by outcome expectancies (individuals understand the potential outcomes of their behavior), self-efficacy (individuals believe that they can achieve their desired behavioral goal), and identification (individuals identify with certain aspects of the role model) (Bandura, 1989). In Gollwitzer’s (1999) theory on Implementation Intentions, individuals are suggested to make plans for anticipated situations, in which their desired behavior is at risk. These plans (implementation intentions) are assumed to delegate the control of goal-directed responses over these critical situations when encountered. Another prominent behavior change theory has been published by Ryan and Deci (2000) and Deci and Ryan (2008). According to their Self-Determination Theory, for behavior change to be successful, three basic psychological needs require fulfillment: autonomy (being the causal agent of one’s own life), competence (ability to master skills important to oneself) and relatedness (feeling connected to others). A number of other theories have each determined a small, inconsistent number of domains supposedly relevant for behavior change initiation.

In a systematic review on 100 behavior change maintenance theories, Kwasnicka et al. (2016) highlight a deficiency in theoretical elaboration regarding the process of maintenance after initial change present in the literature. Theories that are concerned with the behavior change maintenance describe several stages of a behavior change process and the resources necessary to progress from one stage to another. A widely used theory is the Transtheoretical Model (Prochaska et al., 2008), according to which an individual’s change process starts at a precontemplation stage, and continues with the contemplation, planning, implementation, maintenance and termination stages. Similar processes have been suggested by other authors (Weinstein and Sandman, 1992, 2002; Gollwitzer, 1999; Rothman et al., 2004; Schwarzer et al., 2011). For example, Weinstein and Sandman (2002) emphasize the stage before precontemplation where individuals may be unaware of the issue (e.g., that change in diet could improve their health conditions) and Rothman et al.’s (2004) model adds a habit stage where individuals have automated the new behavior. Michaelsen and Esch (2021) have provided the first comprehensive synthesis of behavior change models, a flexible seven-stage behavior change process, which allows to systematically relate motivation and reward mechanisms to these stages. In their process, individuals may experience the stages unawareness, awareness, contemplation, planning, initiation, continued action, and maintenance. These stages are categorized into three phases of engagement, namely, non-engagement, motivational engagement, and executive engagement, in which individuals’ actions are driven by different types of motivation and reward processes (Michaelsen and Esch, 2021, 2022), as explained in more detail below.

## 3. Motivation and reward systems involved in behavior change processes

**FIGURE 1:**
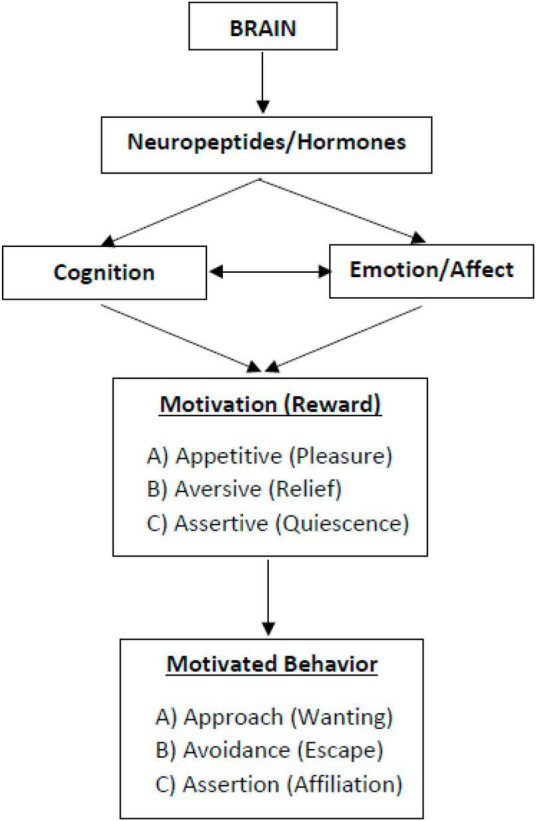
Three types of motivation and reward. Esch (2022); copyright: ©2022 by the author (TE). Licensee MDPI, Basel, Switzerland.

Michaelsen and Esch (2021) have described three types of motivational states (approach motivation, avoidance motivation, and assertion motivation) and their corresponding rewards (pleasure, relief, and quiescence) that seem to play key roles in health behavior change processes (see Figure 1).

### 3.1. Approach motivation

Approach motivation, also known as appetitive or incentive salience, is focused on stimuli or goals that are associated with positive and pleasurable experiences (Bozarth, 1994; Esch and Stefano, 2004; Elliot et al., 2013). This type of motivation is linked to the wanting-system, reward expectation, performance, and action (Esch, 2022). The attainment of a desired stimulus or goal typically produces a sense of pleasure or reward, which may or may not be noticeable depending on the intensity of the experience. The reward is not derived from the stimulus or goal itself, but from the psychological and neurobiological processes that occur when there is a positive anticipation and response to a stimulus or goal (Berridge and Kringelbach, 2008; Schultz, 2015). While it is challenging to categorize experiences into specific types of motivational processes, it is generally agreed that individuals tend to assess stimuli as positive or negative (Elliot et al., 2013). These assessments are frequently referred to as fundamental affective experiences and include emotions such as joy, pleasure, and excitement (Schneirla, 1959; Cacioppo et al., 1999; Elliot et al., 2013; Lang and Bradley, 2013; Rolls, 2013). Therefore, the essence of approach motivation lies in the anticipation of obtaining a reward that is characterized by positive emotions.

The underlying physiological mechanisms of motivation occur in specific brain areas distinct from other sensory and cognitive areas (Kringelbach, 2005; Esch, 2022). The approach motivation and reward system is commonly described as being embedded in the central nervous system (CNS), involves nerve cells that originate in the ventral tegmental area (VTA) and send projections to the frontal brain, specifically the nucleus accumbens (NAC), via the neurotransmitter dopamine (Nestler, 2001; Nestler et al., 2001; Esch and Stefano, 2004, 2010). The nucleus accumbens (NAC) plays a crucial role in the neural regulation of reward-seeking behavior by signaling the degree of effort necessary to acquire a reward and the desire to obtain it, thereby determining the appetitive motivational salience. Additionally, the ventral tegmental area-nucleus accumbens (VTA-NAC) pathway is responsible for measuring and regulating the rewarding aspects of an activity, transmitting pertinent information to other brain regions (Esch and Stefano, 2004; Berridge, 2007; Smith et al., 2011; Esch, 2022). The magnitude of expected reward has been found to significantly influence the likelihood of an individual to retain and repeat a behavior (Esch and Stefano, 2010). Furthermore, the hippocampus and amygdala have been identified as crucial components of the reward system, with the hippocampus serving as a gatekeeper for experiences to be recognized and stored in memory, while the amygdala assesses these experiences as either pleasurable or detrimental (Esch and Stefano, 2004; Nestler and Malenka, 2004). The mesocortical dopamine pathway in the frontal cortex is also known to be involved in the evaluation of the “costs” and risks associated with the pursuit of rewards, ultimately shaping an individual’s behavioral response (Esch and Stefano, 2010).

### 3.2. Avoidance motivation

The construct of avoidance motivation, also referred to as negatively-valenced fearful salience, pertains to the motivational system that drives the avoidance of punishment or potential harm, rather than the pursuit of reward. This type of motivation is intricately linked to the fight-flight-freeze response, which encompasses physiological and behavioral changes in response to perceived threat (Bozarth, 1994; Esch and Stefano, 2004; Seymour et al., 2007; Esch, 2022). The phenomenon commonly known as avoidance behavior is typically evoked by an aversive or challenging stimulus, and elicits a motivated reaction of withdrawal, commonly manifested as the act of moving away from unpleasant conditions. It is noteworthy that avoidance behavior can be differentiated from punishment, which exerts a suppressing effect on the strength of the behavioral response (passive avoidance), and from negative reinforcement, which engenders an augmenting effect on the strength of the behavioral response (active avoidance) (Schultz, 2015). In contrast to active reactions such as fighting or fleeing in response to a fear-inducing stimulus, there can also be the passive reactions of freezing (Berridge, 2018). Emotions associated with avoidance motivation include anxiety, fear, and disgust (Lang, 1995; Cacioppo et al., 1999; Watson et al., 1999; Elliot et al., 2013; Hirschberg and Manning, 2015; Esch, 2022).

Avoidance motivation is embedded in the stress system and involves increased sympathetic activity and the release of cortisol, adrenaline, opioids, and vasopressin (Esch, 2022). This type of motivation is rooted in the lower limbic system, specifically the amygdala and hypothalamus. Upon the anticipation of an actual or imagined threat, two distinct pathways are instigated: one through the hypothalamus and pituitary gland, leading to the release of cortisol, and the other through the sympathetic nervous system, leading to the release of adrenaline (Esch, 2022). The freeze reaction is also connected to the amygdala (LeDoux, 1998). Successful avoidance can lead to relief, a positive, low-arousal emotion that can be experienced as relaxation or reward (Levenson, 2011; Krisam et al., 2017; Esch, 2022). An incontrovertible interdependence between the approach and avoidance motivation systems exists, as akin brain regions are triggered during both relief and other positive affects (Kim et al., 2007; Sangha, 2015).

### 3.3. Assertion motivation

The majority of research on motivation and reward does not differentiate between behavior driven by approach motivation and behavior driven by assertion motivation. In point of fact, these two categories of motivation are frequently confounded or amalgamated (McCall and Singer, 2012), despite the divergent neurobiological mechanisms underlying them, their distinct loci in the brain, and their discrepant behavioral outcomes. Assertion motivation, or assertive salience, is linked to the “non-wanting” system and associated with inaction, acceptance, or contentment, homeostasis, and quiescence. It describes the motivation to maintain a certain condition or state (McCall and Singer, 2012; Esch, 2022). Assertion motivation is different from approach motivation in terms of the emotions it evokes and the types of behavior it leads to McCall and Singer (2012) and Esch (2022). Assertion motivation is associated with a lack of desire to change or move away from the current state, while approach motivation is associated with a desire to move toward something. Assertion motivation can be seen in instances where a person is content with their current situation, such as a newly habituated health behavior, and there is no inclination to change or move away from it.

Assertion motivation is linked to increased activity in the parasympathetic autonomous nervous system and is associated with neurotransmitters such as endogenous opiates, oxytocin, acetylcholine, serotonin, and endocannabinoids (Esch, 2022). Brain areas involved in the activation of assertive motivation include the midbrain, vagus areas, cingulum, hippocampus, ventral striatum, hypothalamus, and pituitary gland (Michaelsen and Esch, 2021). It is different from approach and avoidance motivation in terms of related affective qualities and behavioral outcomes and is not characterized by activation of dopaminergic activity.

## 4. Weaving together motivation and reward mechanisms with health behavior change theories

Weaving together psychological explanations of behavior change with neurobiological understandings of motivation and reward processes has produced three models explaining different aspects of behavior change. First, a model differentiating goal-directed and stimulus-driven behavior (Michaelsen and Esch, 2021) will be explained. This is followed by the description of the Model of Engagement, that illustrates the role of the three types of motivation during a behavior change process (Michaelsen and Esch, 2021). Finally, the behavior change resource model (Michaelsen and Esch, 2022) that integrates the differentiation between goal-directed and stimulus-driven behavior with the Model of Engagement to explain the functional mechanisms of behavior change techniques is presented. The elaborations of Ludwig et al. (2020) concerning reward valuation and Letzen et al. (2019) on mesocorticolimbic function in behavior change are discussed within these sections.

### 4.1. Goal-directed and stimulus-driven behavior

Kwasnicka et al.’s (2016) systematic review revealed that existing health behavior change theories largely focus on cognitive resources deemed necessary for achieving behavior change. Their findings indicated that only 10% of the theories reviewed took into account the relevance of automatic responses to relevant cues or stimuli, which has been identified as a limitation to existing theories (Van Cappellen et al., 2018). This is because the manifestation of health behaviors in daily life is often influenced by implicit emotions and non-cognitive motives, rather than reflective cognitive willpower, as various dual-process models have emphasized (e.g., Kahneman and Tversky, 1982; Strack and Deutsch, 2004; Hall and Fong, 2007; Marteau et al., 2012; Sheeran et al., 2013). Dual-process models of decision-making have been developed to differentiate between two regulatory systems in the brain: reflective (cognitive, conscious) and affective (impulsive, intuitive, automatic) antecedents of behavior (Chaiken, 1980; Petty and Cacioppo, 2012). The reflective system is based on conscious deliberation and control, which requires subjective effort. It draws upon an individual’s knowledge of probabilities and values and is based on rules of language and logic. The key processes of the reflective system are volition and reasoning, which can be intentionally accessed. However, the reflective process is relatively slow (Strack and Deutsch, 2004; Sheeran et al., 2013). The reflective system typically supersedes the automatic system, which is quicker and more effortless, and operates by utilizing stored associations acquired through experiences, responding to habits and impulses. Strack and Deutsch (2004) posit that the automatic system is a significant impulsive process that engenders activation, in which perceptual inputs stimulate elements in the associative memory, subsequently activating other related elements. This form of information processing is characterized by its rapidity and operation beyond conscious awareness, as noted in the extant literature (Strack and Deutsch, 2004; Evans, 2010; Sheeran et al., 2013). While this view has garnered both commendation and condemnation from scholars (Evans, 2018), it nevertheless represents a significant contribution to the comprehension of health behavior and behavior change. Furthermore, a widespread view stemming from dual-process models is that the more rapid component governs behavior.

**FIGURE 2:**
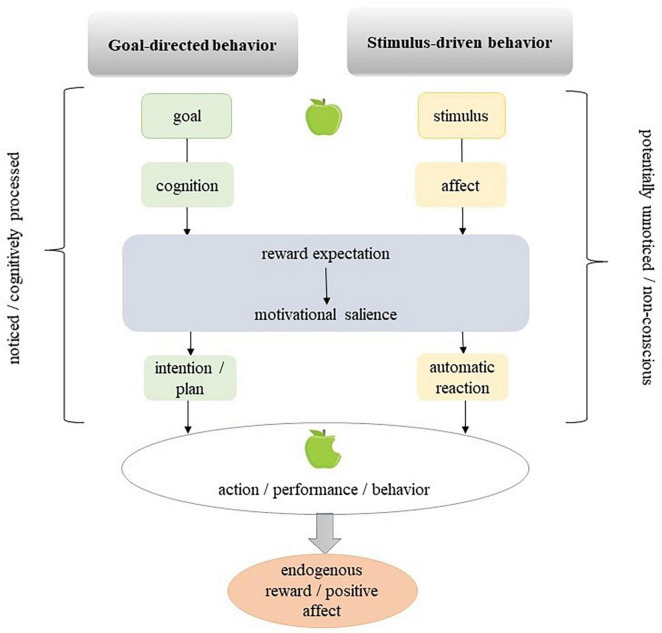
Goal-directed and stimulus-driven behavior (Michaelsen and Esch, 2021).

In reference to dual-process models and the differentiation between controlled goals and autonomous goals (or unnoticed stimuli), Michaelsen and Esch (2021) present a neurobiologically informed model of stimulus-driven and goal-directed behavior. In stimulus-driven behavior, a stimulus activates automatic processes and leads to behavior without the individual having noticed the stimulus. Once a stimulus has undergone cognitive processing and been transformed into a goal, the ensuing behavior is referred to as goal-directed behavior. The authors posit that both varieties of stimuli are capable of inciting appetitive, aversive, or assertive salience by means of reward anticipation. In this way, motivational salience, or the ability to attract and hold attention, can lead to action and engagement without conscious thought or planning (Ryan and Deci, 2000; Carver, 2009; Kruglanski et al., 2014; Berridge, 2018). Both unnoticed stimuli and those that are deliberately processed can result in the same actions and engagement. However, in goal-directed behavior, the individual is aware of their actions and is actively involved in the process, as noted by Michaelsen and Esch (2021). Figure 2 illustrates the difference between stimulus-driven and goal-directed behavior in a simplified way.

The model can be expanded by the theory proposed by Ludwig et al. (2020), who propose an approach to achieve sustainable behavior change through a combination of theories and research on autonomous motivation, reinforcement learning and mindfulness. The authors argue that behavior change can occur through increased awareness of the reward value of specific actions, which drives future behavior, in addition to the commonly proposed “mental gap” mechanism. The stability of a behavior depends on changes in its reward value over time and the accessibility of more rewarding behaviors. The reward value of a behavior may depend on both external and internal factors, such as subjective experience and goal achievement. The authors suggest that bringing present-moment or mindful awareness to current behavior can update the reward value of habitual behaviors and lead to new learning. This approach involves direct, in-the-moment, curious awareness and is not reliant on reflective thought processes. An increased awareness about stimuli that engender change through increased reward value would shift individuals, in the above model, from stimulus-driven to goal-directed behavior.

### 4.2. Motivational engagement in behavior change processes

Based on the synthesis of multi-stage behavior change theories, Michaelsen and Esch (2021) have derived three different phases of engagement, based on the role of motivational processes involved during the stages of behavior change. During the first phase, called non-engagement phase, individuals are either unaware that behavior change may improve their health conditions, or they are aware but have no intention to change an aspect of their health behavior. During this phase, any motivational mechanisms are yet absent. Stimuli like new information about the health benefits of a certain behavior change may activate motivational processes so that individuals progress into the motivational engagement phase, which is comprised of the contemplation and planning stages.

The nature of the contemplative phase is contingent on the sort of motivational salience that is evoked by the stimulus. Should an individual be satisfied with their present state, assertive salience becomes operational. Here, the likelihood of perpetuating the present condition is linked to positive valence that instigates sensations of quiescence, stillness, and/or relaxation stemming from the discharge, such as that of endogenous opiates, oxytocin and related neurotransmitters, as well as parasympathetic activity. Such a state leads to a lack of behavioral activity, resulting in the cessation of the process of behavior change. In the event that an individual desires a change, either appetitive or aversive salience is elicited. When appetitive salience is activated, information undergoes processing by the mesocortical dopamine pathway in the frontal cortex, and a preference for a new behavior is set (Esch and Stefano, 2010; Michaelsen and Esch, 2021). On the other hand, should aversive salience be activated, information is routed through the stress response pathways, namely, the hypothalamic-pituitary (-adrenal) axis and the (amygdalar-) sympathetic nervous system axis (Esch and Stefano, 2010).

**FIGURE 3:**
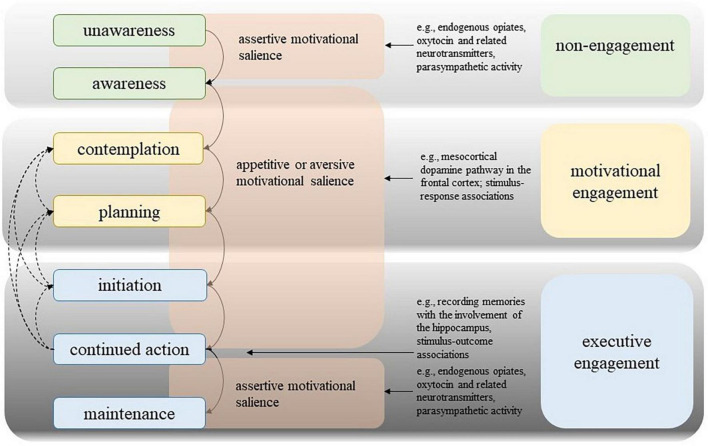
Model of Engagement (Michaelsen and Esch, 2021).

The planning stage is defined by cognitive, goal-directed action (see Figure 3). In order to plan, the actions of thinking, reflecting, and evaluating are involved, and, neurobiologically, the upper limbic level. The cognitive task of planning is propelled by either appetitive or aversive motivational salience and may culminate in an intention, or a series of intentions (a plan). Michaelsen and Esch (2021) contend that, owing to its cognitive underpinnings, planning can only transpire in goal-directed behavioral processes, and not in stimulus-driven behavioral processes. They posit that both stages of motivational engagement can be bypassed if the presented stimulus and the evoked motivational salience go unnoticed (i.e., are stimulus-driven).

The third engagement phase is called executive engagement and consists of the stages initiation, continued action and maintenance (Michaelsen and Esch, 2021). According to the authors, initiation is the behavioral consequence of a response-outcome mechanism, whereby an individual actively reacts to the appetitive or aversive motivational salience that ensues from the encounter (and processing) of a stimulus. This reaction is propelled by the anticipation of pleasurable feelings (in the case of positive stimuli) or relief (in the case of negative stimuli). The appraisal of experiences as pleasurable or unpleasurable takes place within the endogenous reward system (such as the amygdala), which also encompasses the establishment of associations between an experience and other stimuli (Michaelsen and Esch, 2021). Upon fulfillment of the expectation of a positive experience, said experience engenders a memory that, in turn, spawns an anticipation of a reward from the same activity, thereby enhancing the likelihood of the behavior being reiterated (Van Cappellen et al., 2018). This phenomenon is referred to as reward responsiveness (Carver and White, 1994).

The process of recording memories of experiences, which includes the context in which they occurred, such as the location, time, and social companionship, entails the involvement of the hippocampus (Nestler, 2001; Nestler et al., 2001; Esch and Stefano, 2004, 2010). This type of learning can lead to a reciprocal effect: as time passes, associations between positive affect and stimuli that predict it, and memories of it, may endow those stimuli with appetitive salience, making them more likely to capture attention in the future (Fredrickson and Joiner, 2018; Van Cappellen et al., 2018). The phenomenon of learning encompasses two critical components, namely conditioning and expectation. In the context of stimulus-driven and goal-directed behavior, the experience of reward is not contingent on whether the stimulus was subjected to cognitive processing to be transformed into a goal. According to Michaelsen and Esch (2021), the initiation of a new behavior through the activation of endogenous reward triggers a learning process, wherein the association between the new behavior and the experienced positive affect fosters reward expectancy, potentially resulting in continued action. The present study posits that the maintenance of response-outcome associations between pleasurable stimuli and their predictive cues is enhanced by sustained behavioral engagement. In this context, the authors assert that the probability of repetitive behavior, and consequently the degree of engagement therein, is contingent upon the magnitude of endogenous reward elicited by the new behavior (Michaelsen and Esch, 2021). Following the repeated enactment of stimulus-driven or goal-directed behavioral actions, individuals ultimately transition into a maintenance stage, characterized by a sustained operant learning process that leads to habit formation (Schultz, 2015). During this stage, the behavior is executed with regularity, and the assertive salience driven by the motivation and reward systems remains active, thereby strengthening the habitually performed action (Michaelsen and Esch, 2021). The experience of quiescence, calm or contentment associated with the activation of the parasympathetic nervous system and other down-regulatory pathways serves as a powerful motivator for the maintenance of newly adopted behaviors. This state of contentment engenders a state of “non-wanting” with regard to further modifications of behavior (Michaelsen and Esch, 2021). This Model of Engagement is presented in Figure 3.

The findings can be integrated with the idea of Letzen et al. (2019), who incorporate putative neurobiological mechanisms contributing to motivation for pain self-management into the Motivational Model for Pain Self-Management (Jensen et al., 2003). The authors propose that an altered function in the mesocorticolimbic function would inhibit behavior change. The goal of this updated model is to determine whether potential neurobiological deficiencies contributing to poor motivation feed into observed non-adherence among patients with chronic pain. The authors hypothesize that mesocorticolimbic function subserves treatment-related learning history, contingency processing, and cost/benefit analysis, and individuals with mesocorticolimbic dysfunction will have lower perceived importance of symptom self-management and poorer self-efficacy for symptom self-management. They also suggest that magnitude of mesocorticolimbic dysfunction will correlate with reported treatment motivation, so that greater dysfunction is associated with poorer readiness for change, and that self-reported treatment motivation moderates the relationship between pre-treatment mesocorticolimbic function and adherence (Letzen et al., 2019). The article also suggests that practice of a pain management strategy will be associated with mesocorticolimbic activity via reinforcement, and individuals with high reinforcement from this practice will have greater motivation for future practice, leading to better adherence (Letzen et al., 2019). While the authors do not discuss pain management behavior as a process, by relating their hypothesis to the Model of Engagement, we can derive that mesocorticolimbic dysfunction would inhibit the progress to the stages contemplation, planning, initiation and/or continued action, and individuals with mesocorticolimbic dysfunction facing these stages within their health behavior change process would need specific support to progress.

### 4.3. The behavior change resource model

#### 4.3.1. Three types of behavior change resources

The resources individuals need to progress from one health behavior change stage to another, as suggested in a number of health behavior change theories, have been summarized by Cane et al. (2012), Kwasnicka et al. (2016), and Carey et al. (2019). Recently, the resources that facilitate changes in health behavior have been classified by Michaelsen and Esch (2022) into two broad categories, namely the socio-environmental resources external to the individual, and the bio-psychological resources that pertain to the internal state of the individual, with both types being characterized by changeable and non-changeable factors. While behavior change techniques (BCTs) cannot be leveraged to address non-changeable factors such as the weather, their utility is geared to targeting changeable resources (Michaelsen and Esch, 2022).

**FIGURE 4:**
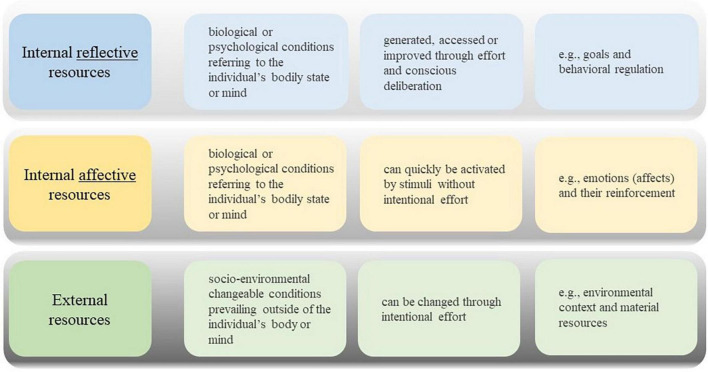
Three types of changeable resources (Michaelsen and Esch, 2022).

Based on the distinction between reflective and affective aspects, Michaelsen and Esch (2022) have established a categorization of resources according to how these resources are accessed or generated in the brain. As such, resources are either external (socio-environmental), or internal (bio-psychological), whereby the latter can be either reflective or affective. Reflective resources are accessed, generated or refined through deliberate and effortful cognitive processing, including but not limited to goal-setting and behavioral regulation. In contrast, affective resources, such as emotions and their reinforcing valences, may be promptly elicited by environmental stimuli without the need for volitional engagement. External resources, such as environmental context and material resources, can be externally provided (Michaelsen and Esch, 2022). These three types of changeable resources are depicted in Figure 4.

#### 4.3.2. Behavior change techniques

Behavior change theories provide a foundation for developing effective behavior change techniques (BCTs) to support individuals in modifying their behaviors. Such theories have been employed in diverse ways, including the integration of social interactions based on Bandura’s (1989) Social Cognitive Theory, and assisting patients in generating implementation intentions, drawing on Gollwitzer’s (1999) theory on Implementation Intentions [see Bélanger-Gravel et al. (2013) for a meta-analysis of BCTs based on Gollwitzer’s (1999) theory on Implementation Intentions]. The extant literature has primarily focused on employing behavior change techniques (BCTs) that enhance cognitive resources, such as nutritional or psychological counseling (Ball et al., 2013), or create situations that promote behavior modification, such as supervised walking groups (Kassavou et al., 2013) or financial incentives (Lee et al., 2019). However, these techniques often fail to account for patients’ individual differences in needs and circumstances (Cecchini et al., 2010). The majority of interventions geared toward behavioral change tend to be financially costly and hence, not sustainable over a prolonged period of time or feasible to offer to a wide populace (Forster et al., 2011). Some interventions have also yielded adverse side effects. For instance, monetary rewards for weight loss have been shown to be effective until the remuneration is obtained; however, subsequent weeks have reported higher odds of weight gain (Paul-Ebhohimhen and Avenell, 2008). In contrast to BCTs that mainly, or solely, address cognitive, rational, or circumstantial/environmental resources and domains, modern BCTs primarily build on individual behavioral responses to various motivational stimuli, including affective components of a behavioral decision. Examples are the use of wearables (e.g., Piwek et al., 2016) and other digital innovations (e.g., Priesterroth et al., 2019) as well as reminders (e.g., Orr and King, 2015) among various forms of nudging. Nudging can be understood as shaping decision contexts in a way that encourages a particular behavior (e.g., Hansen and Jespersen, 2013) in a playful way through the activation of affective processes in the brain (Michaelsen and Esch, 2022).

Despite a rapid growth in the implementation of interventions, most of these interventions are only successful in the short term, and often fail to demonstrate a significant improvement in the medium and long term (e.g., Marteau et al., 2012; International Diabetes Federation [IDF], 2013; Ulrich et al., 2016; Sainsbury et al., 2019). One reason for this may be the lack of comprehensive theories that allow developing successful BCTs. Another reason may be the insufficient use of theories in intervention development. In a scoping review pertaining to nudging interventions, it was discovered that only a quarter of the studies under review took into consideration the purported working mechanisms underlying the effectiveness of the intervention, while three-quarters focused solely on demonstrating its efficacy (Szaszi et al., 2018). The working mechanisms, which involve the connections between BCTs and the targeted domains or resources, i.e., the specific BCT that addresses a particular resource, were elucidated upon by Carey et al. (2019). A detailed list of resources relevant to behavior change initiation is presented by Michie et al. (2005), who identified 112 behavior change theories and clustered the domains of behavior change mentioned therein into 12 categories. This Theoretical Domains Framework has been validated by Cane et al. (2012), who extended the number of categories to 14 domains: “knowledge,” “skills,” “social/professional role and identity,” “beliefs about capabilities,” “optimism,” “beliefs about consequences,” “reinforcement,” “intentions,” “goals,” “memory,” “attention and decision processes,” “environmental context and resources,” “social influences,” “emotion,” and “self-regulation”. Kwasnicka et al. (2016) have summarized the domains that have been presented relevant for behavior change maintenance in their reviewed maintenance theories into five overarching categories; “maintenance motives,” “self-regulation,” “resources,” “habit,” and “environmental and social influences.” These inhibit significant overlaps with Cane et al.’s (2012) 14 domains. An analysis of these resources and the BCTs they are targeted by is presented by Michaelsen and Esch (2022), as is further explained below.

#### 4.3.3. Clustering BCTs

Based on the triad of behavior change resources, BCTs can be clustered according to how they address these resources and can thereby be described as the functional mechanisms of BCTs. In this way, Michaelsen and Esch (2022) derived three types of BCTs, namely those, that provide external resources (facilitating), those which strengthen internal reflective resources (boosting) and those that activate internal affective resources (nudging).

##### 4.3.3.1. Facilitating

BCTs that focus on providing external resources enable individuals to engage in a desired behavior. These resources, which fall under categories such as “environmental context and resources” and “social influences” in the Theoretical Domains Framework (Michie et al., 2005; Cane et al., 2012), can be provided by the individual, another person, or an organization. Illustrative of the aforementioned interventions are strategies that enhance the availability of healthy food alternatives within workplace canteens (Geaney et al., 2013), incentivization programs that offer monetary rewards (Petry et al., 2013), modification of the physical environment through initiatives such as the establishment of public fitness trails (Cohen et al., 2012), and social support mechanisms including the facilitation of assisted walking groups (Kassavou et al., 2013). These techniques can help facilitate behavior change, but the new behavior may not be sustained once the external resources are removed. However, when an individual has established a routine or habit of a specific new behavior, and their motivation to continue is strong, the end of the availability of the BCT may lead to a similar behavior that can be implemented independently of the original BCT. As an example, the termination of an organized walking group may prompt the participants to either sustain their walking activity on an individual basis or establish autonomous walking groups.

##### 4.3.3.2. Boosting

Internal reflective resources can be addressed by involving cognitive processes. BCTs which target theses resources are called boosts. These enjoyable tasks foster the building up or strengthening of internal reflective resources that can support health behavior change. Examples are “beliefs about capabilities,” “beliefs about consequences,” “intentions,” “goals,” and “behavioral regulation” (Cane et al., 2012). These types of interventions may include self-monitoring techniques, such as keeping a diary or practicing mindfulness (Shomaker et al., 2019) to improve attention and awareness. Additionally, interventions like health education (Gigerenzer et al., 2007) and nutritional counseling (Ball et al., 2013) can increase an individual’s understanding of the consequences of their behavior and lead to a willingness to change. There are also other examples of boosting interventions (see, e.g., Grüne-Yanoff and Hertwig, 2016) that can similarly lead to an increased readiness to change and intentional implementation of a desired behavior (Michaelsen and Esch, 2022). Having executed the desired behavior by means of one’s own effort, thus, leads to an experience of self-efficacy and the related positive affect. This in turn, can act as a reinforcement to pursue the behavior again. The generated effects potentially persist beyond the intervention, if those resources have become sufficiently strong or stable (Hertwig and Grüne-Yanoff, 2017) and the reward, e.g., through the self-efficacy experience, has been sufficiently intense and therefore been stored in memory.

##### 4.3.3.3. Nudging

Nudges are interventions that guide people toward a certain behavior without limiting their freedom of choice (e.g., Thaler and Sunstein, 2008; Alemanno and Sibony, 2015; Halpern and Sanders, 2016). This is achieved by manipulating aspects of the environment to create cues, stimuli, or triggers that make the desired behavior more appealing. Nudging activates the emotional aspects of decision-making, making the behavior more attractive, enjoyable and intrinsically rewarding, while still allowing individuals to make their own choices (Michaelsen and Esch, 2021, 2022). Nudging does not require cognitive skills or external resources, but it activates non-conscious or automatic resources to compensate for the lack of external or reflective resources needed for behavior change (van Gestel et al., 2020). Felsen and Reiner (2015) provided a neuroscientific explanation of how nudges exert their effects based on diffusion-to-bound models. In diffusion-to-bound models, it is assumed that a decision is made within a decision space bounded by the available choices. A decision variable that is comprised of multiple factors that influence the decision including current sensory stimulation, stored memory about past experience, and the subjective value of each option, moves further or closer to each bound depending on the strength of these factors until one bound is reached and the corresponding decision is made (Felsen and Reiner, 2015). Nudges can be considered to shift the decision variable toward the bound of the preferred choice, i.e., making the preferred choice more likely (Felsen and Reiner, 2015).

In a systematic review, nudging interventions have been shown to lead to medium size effects in behavior change (Mertens et al., 2022). Examples are variations in the manner of presenting food items (Bucher et al., 2016; Broers et al., 2017; van Gestel et al., 2020), reminders or reinforcement-based learning schemes (Orr and King, 2015; Yom-Tov et al., 2017), lotteries (Volpp et al., 2008), and point systems (Priesterroth et al., 2019), all of which serve to augment the expectation of rewards. The underlying premise is that the magnitude of the anticipated reward is positively correlated with the likelihood of remembering and repeating it (Esch and Stefano, 2004). These nudges are believed to only have temporary effects on behavior, as the increased motivation from the nudge is not sustained once the nudge is removed. For example, a study that used point-of-decision prompts to encourage stair use in a university dormitory found that the effects were not sustained once the prompts were removed (Howie and Young, 2011). However, with frequent repetition, the behavior being nudged may become a habit that continues even after the nudge is removed because of neurobiological learning processes (Verplanken and Aarts, 1999; Lieberoth et al., 2018; van Rookhuijzen et al., 2021).

#### 4.3.4. Summary of the behavior change resource model

**FIGURE 5:**
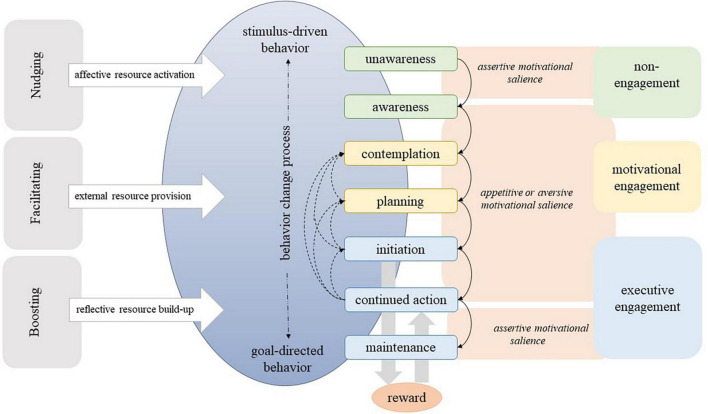
Behavior change resource model and its relation to the Model of Engagement (Michaelsen and Esch, 2021, 2022).

The classification of BCTs based on the behavior change resources they address, may be sufficient to define all existing BCTs and explain their functional mechanism. This means that any BCT, such as those listed in Michie et al. (2013) can be categorized as facilitating, boosting, or nudging. Michaelsen and Esch (2022) have defined resource-driven behavior change as a process that increases the likelihood of a preferred behavior by focusing on the resources needed for that particular behavior to occur. Resource-driven behavior change is accomplished via the implementation of one or a blend of three BCT types that provide external resources (facilitating), build up internal reflective resources (boosting) or activate internal affective resources (nudging). Upon achieving a certain level of efficacy, the BCTs can prompt the initiation or maintenance of a new behavior, which can subsequently yield a positive response (affect) as a reward. Such reward can serve as a cue or stimulus to augment resources, known as vantage resources (Van Cappellen et al., 2018). Exemplifying this notion, a positive affect can function as a reinforcement, thereby acting as a subtle prompting mechanism (nudging), as the experience of a pleasurable affect is deemed vital in predicting the likelihood of subsequent behavioral engagement (Michaelsen and Esch, 2022). Furthermore, successful implementation or repetition of the desired behavior can also reinforce other desirable cognitive and affective states, such as strengthening one’s belief in one’s own abilities (i.e., self-efficacy), which can serve as a boosting strategy. Neurobiologically, these emotional influences on reward experiences and subsequent decisions are mediated in the medial prefrontal cortex, as evidence from human and animal model studies indicates (Euston et al., 2012). Therefore, the functional mechanisms of BCTs are not independent, but interrelated with neurobiological motivation and reward proceedings. Recognizing these multidirectional causal relationships, Michaelsen and Esch (2022) propose a new framework for understanding the functional mechanisms of BCTs, called the behavior change resource model (BCRM). The BCRM and its relation to the Model of Engagement is illustrated in Figure 5.

## 5. Discussion

### 5.1. Understanding health behavior change by motivation and reward mechanisms

Despite being essential to enhance health, behavior change support is rarely covered by health care systems around the world (Chauhan et al., 2017; Grabovac et al., 2019). It is therefore even more important to support the development of interventions, which are powerful in terms of efficiency and preservation of individuals’ autonomy in order to be applied in low-resource settings or independently of political decision-makers. Behavior change has been studied primarily from a social psychology perspective, focusing on cognitive, or reflective, resources and domains relevant to behavior change, including circumstantial/environmental aspects. Neurobiological advances in automatic functioning as well as motivation and reward systems, however, fit neatly into the discussion of how humans act and how behavior can be changed. Integrating motivation and reward mechanisms into the behavior change literature and presenting new models to understand behavior change potentially helps policy makers to identify the necessary and sufficient environmental, economic, and psychological conditions that make healthy choices possible and easy.

A framework with a similar purpose as the behavior change resource model is the behavior change wheel (BCW) (Michie et al., 2011). The BCW is based on a review of 19 behavior change frameworks from various fields (e.g., health, environmental behavior). Its core is a “behavior system” with three essential conditions: capability, opportunity, and motivation. These three conditions can be interpreted as attributes of behavior change resources. They overlap slightly with the categorization of resources made by Michaelsen and Esch (2022), in the sense that opportunity to behavior change is present when external resources are available, capability is fulfilled when the necessary internal reflective resources are strong enough, and motivation can be seen as an internal affective resource. In the BCW, motivation represents a psychological resource (referring to intrinsic vs. extrinsic motivation) and is not discussed or integrated in terms of its neurobiological underpinnings. In a second step, Michie et al. (2011) developed intervention functions, which are essentially a categorization of BCTs into nine groups: education, persuasion, incentivization, coercion, training, restriction, environmental restructuring, modeling and enablement. The three conditions are then linked to the intervention’s functions without a specific explanation of how the conditions relate to the interventions, i.e., their functional mechanisms are not explained.

### 5.2. Practical implications of the presented literature

The understanding of the role of motivational salience in health behavior change processes presented by the Model of Engagement could be applied to develop suitable cues and stimuli, e.g., nudges that direct people’s actions into their desired outcome. General examples are fruit placement experiments (Wansink et al., 2011; Hansen et al., 2016) or goal formation through social comparison (Custers and Aarts, 2005, 2010). The findings can also be used in a more differentiated way. Considering the seven stages of the behavior change process, findings imply that different BCTs are required depending on where at the change process an individual is. At the unawareness stage, individuals are not aware that behavior change could contribute to their health status. Therefore, to move to the next stage, individuals require knowledge, insight or possibly a shift in health locus of control from external to more internal (i.e., perceiving the reward from one’s behavior as contingent on one’s own behavior, see Rotter, 1966, or Cheng et al., 2016 for a meta-analysis on health locus of control and specific health behaviors). Knowledge can be provided, for example, through large-scale policy campaigns. Once an individual is aware that a change in behavior could positively affect their current or future health, a number of other resources may be required to spike interest in behavior change and to move into the motivational engagement phase. For example, hearing or reading about personal experiences from peers (e.g., friends, colleagues) could lead to goal formation. Thus, an individual could be incentivized to talk with peers about their health behavior goals and achievements. To move from the contemplation to the planning stage, information about various offers of health promotion courses could be beneficial. In general, the findings can be applied using these three steps:

1. Determining at which stage of their individual change process an individual is.
2. Identifying the resource(s) needed to reach the next relevant stage.
3. Selecting a BCT that targets the lacking, weak or inactive resource.

Step 1 can be done by applying motivational interviewing (Rollnick and Miller, 1995) or the set of questions developed by Michie et al. (2005). For step 2, the Theoretical Domains Framework by Cane et al. (2012) or any other framework that lists health behavior change resources, can be used. Once the lacking, weak or inactive resources for successful behavior change have been identified, one or more suitable BCTs can be selected and applied (step 3). Michaelsen and Esch (2022) provide guidance for the third step in their application guide of the BCRM. In this table, the potential target groups for each type of BCT, based on the seven-stage behavior change process described above, are explained and numerous examples for various settings and stakeholders are given. This can assist health/behavior therapists, intervention planners and patients in selecting appropriate measures to achieve the desired health behavior change.

From a public health perspective, the findings of the studies can also contribute to improve health literacy of specific patient groups, e.g., the chronically ill, or specific populations, such as vulnerable families, e.g., in low-income settings. By identifying the needs of these groups in relation to their health, knowing which type of motivation to foster and which resource to provide, strengthen, or activate with which measures, health behavior (e.g., diet) and disease management (e.g., regularly measuring blood sugar) can be improved. Thus, the findings can also be used in prevention and health promotion contexts and potentially help to close the gap in life expectancy between low- and high-income communities.

Furthermore, the findings have the potential to improve intervention effectiveness by better matching the goals of the intervention and the goals of the patients or individuals for whom they are developed. Interventions with a better fit promise better outcomes (Michie and Prestwich, 2010; Prestwich et al., 2014; Beard et al., 2019; Carey et al., 2019) and could therefore be more cost-effective, thereby relying less on scarce financial resources of providers, such as health insurances, local governments, or states.

### 5.3. Avenues for future research

The results presented in this review are theoretical in nature and therefore require empirical verification. In addition, a number of aspects contained in the studies need to be explored further or discussed in more detail. Some of these points for future research are highlighted in the following.

First, for a number of research strands processed in this review, systematic rather than convenience literature searches could help to substantiate the claims made. While systematic literature searches have been conducted and reviews published on behavior change resources and BCTs (e.g., Cane et al., 2012; Prestwich et al., 2014; Kwasnicka et al., 2016), as well as on behavior change frameworks that served as a basis in the presented analyses (e.g., Kwasnicka et al., 2016; Carey et al., 2019), conducting new systematic searches and reviews could help to integrate the knowledge gained since the reviews were published. Especially the growing literature on single- or multi-system models (as alternatives to dual-process models) of behavior would benefit from a systematic overview and discussion of the advantages and disadvantages of the views published so far. On this basis, the BCRM potentially requires refinement. The literature on health promotion and behavior change is growing rapidly, so more up-to-date reviews could help to increase the granularity and accuracy of the findings. For example, a future systematic search of the behavior change resource literature could be done to map the resources identified in the literature to the three types of resources generated in this review. A comprehensive list of internal affective, internal reflective and external resources could be the result. This list could then be augmented by neurobiological analyses of the functional mechanisms proposed. In addition, a systematic search and analysis of empirically tested BCTs can result in a list and discussion of BCTs in the light of their functional mechanisms. Providing such lists would facilitate the application of the BCRM such that users could easily identify resources they need and the BCTs that help to address them.

Second, the Model of Engagement could be tested in the “real world” with patients, e.g., in primary care settings, through interviews that help patients to describe how they perceive their own behavior change process, at which stage they assume to be and what they require to move forwards. These descriptions are presumably very diverse and depend on patient characteristics, such as age, disease or cultural background. Qualitative interviews are potentially the right starting point for the development of a more general questionnaire to test the model in a larger population and with specific target groups.

Third, the BCRM, and aspects of it, is proposed based on several implicit hypotheses that need empirical verification. The first hypothesis is that it is possible to determine uniquely the stage in the behavior change process for each individual patient. This hypothesis could be tested through a new questionnaire that builds upon motivational interviewing (Rollnick and Miller, 1995) or the set of questions developed by Michie et al. (2005) with specific reference to the stages developed in the Model of Engagement. The second hypothesis is that motivation and reward systems are required to process along the stages. This could be tested by interviewing individuals who have successfully progressed along their stage process with respect to their own description of their affective states (pleasure, relief, quiescence) that were present while progressing. The third hypothesis is that resources can uniquely be classified into internal affective, internal reflective and external resources. Neuroscientific methods such as brain imaging could be used to analyze the affective and motivational components associated with these resources and potentially involved in various BCTs. The fourth hypothesis is that certain BCTs influence resources through the three described functional mechanisms of facilitating, boosting, or nudging. Qualitative research methodologies may provide a means to expound upon the perceptions of individuals who have undergone specific interventions, in relation to the mechanisms that either support or impede their engagement in behavior change processes.

Future research should explore the specific functional mechanisms of BCTs in more detail. So far, the literature presents only a general understanding of the functional mechanisms of BCTs. The BCRM should be subject to further scrutiny by investigating the intricate affective processes that underlie nudging interventions, through the assessment of affective states before, during, and after decision-making. Gaining insight into the neurobiological mechanisms that underpin the three functional components of the BCRM, and their respective roles in determining motivational salience and reward intensity, would undoubtedly enhance the scientific knowledge base and prove invaluable in the development and implementation of future interventions in everyday settings.

Finally, the application process of the three steps with patients or communities could be accompanied by research on its applicability, feasibility and effectiveness to optimize the model and its features for future use.

## 6. Conclusion

Previous theories of health behavior change have overemphasized either cognitive, rational, or relational aspects, while largely neglecting the emotional-affective or motivational processes involved in behavior change. Recent literature has integrated neuroscientific evidence and evidence-informed models into the explanations of how health behavior can be changed, short-term and long-term. Thereby, classifications of behavior change resources and behavior change techniques have been developed and the mechanisms of behavior change techniques have been explained. All in all, the literature has potential to be enriched by more neuroscientific evidence, e.g., more details of the functional mechanisms of health behavior change techniques for particular behavior change resources. Other interesting avenues for future research have been described in this review.

## Author contributions

MM was responsible for initial literature search, article screening, interpretation of the existing research, conducting the analysis, as well as writing, and critical revision of the manuscript. TE provided support from the idea through the conception and design of the review and also provided suggestions for revising the manuscript. Both authors contributed to the article and approved the submitted version.
